# On-Line Survey About Autonomic Dysreflexia in Individuals with Spinal Cord Injury in Croatia

**DOI:** 10.3390/jcm14030670

**Published:** 2025-01-21

**Authors:** Dean Markić, Željka Minić, Josip Šimičić, Karin Kuljanić, Josip Strčić, David Bonifačić, Ivan Marin Sušanj, Ante Jakšić, Helena Sveško Visentin, Robert Ehrman, Marin Marinović

**Affiliations:** 1 Department of Urology, Clinical Hospital Center Rijeka, 51000 Rijeka, Croatia; dean.markic@medri.uniri.hr (D.M.); ramm.ivanmarin@gmail.com (I.M.S.); 2 Faculty of Medicine, University of Rijeka, 51000 Rijeka, Croatia; 3 Faculty of Biotechnology and Drug Development, University of Rijeka, 51000 Rijeka, Croatia; zeljka.minic@biotech.uniri.hr; 4 Department of Emergency Medicine, School of Medicine, Wayne State University, Detroit, MI 48201, USA; rehrman@wayne.edu; 5 Adriainvest Engineering, 10000 Zagreb, Croatia; josip.simicic@gmail.com; 6 Department of Psychology, Clinical Hospital Center Rijeka, 51000 Rijeka, Croatia; kkuljanic@gmail.com (K.K.); helenasvesko@gmail.com (H.S.V.); 7 Faculty of Health Studies, University of Rijeka, 51000 Rijeka, Croatia; 8 Department of Emergency Medicine, Clinical Hospital Center Rijeka, 51000 Rijeka, Croatia; jstrcic@unicath.hr; 9 Department of Nursing, Catholic University of Croatia, 10000 Zagreb, Croatia; 10 Department of Neurology, Clinical Hospital Center Rijeka, 51000 Rijeka, Croatia; david.bonifacic@medri.uniri.hr; 11 Department of Surgery, Clinical Hospital Center Rijeka, 51000 Rijeka, Croatia; marin.marinovic1@medri.uniri.hr

**Keywords:** autonomic dysreflexia, spinal cord injury, quality of life, intermittent self-catheterization

## Abstract

**Background/Objectives:** Autonomic dysreflexia (AD) is a clinical syndrome affecting persons with spinal cord injury (SCI). The aim of the study was to present the experience of individuals with SCI and AD in Croatia. Single-center questionnaire study. Setting: Faculty of Medicine, University of Rijeka, Rijeka, Croatia. Persons with SCI were divided into those with an SCI at T6 and above (*n* = 41) and those with an SCI at T7 and below (*n* = 29). Based on anamnestic data, patients with an SCI at T6 and above were further divided into those with and without self-reported clinical symptoms of AD (respectively: *n* = 33 and *n* = 8). **Methods:** The online survey included 23 questions of various types. It consisted of a general (demographic) section, section with specific questions about AD, and a section on self-assessment and quality of life. Based on the answers, the experience and knowledge of AD among individuals with an SCI in Croatia was assessed. **Results:** A total of 70 individuals with an SCI completed the whole survey. The average age was 40.6 years. The patients were predominantly male (72.9%) and mostly with a traumatic SCI (84.3%). Of the 70 individuals with an SCI, 35 (50%) reported they were familiar with AD, with the majority indicating that the internet was their primary source of information. Among those with an SCI who were unaware of AD, most (34/35 = 97.1%) wanted to learn about AD. The self-assessed quality of life was insignificantly better in patients with an SCI below T6 than in those with higher lesions. Patients with AD reported different symptoms and the most frequently reported symptom was a headache (70%). In 64% of participants, the primary trigger of AD were issues with the urinary bladder. In most individuals (55%), symptoms were not recognized by the healthcare providers. The individuals with AD were dissatisfied with the information about AD they received from doctors or nurses. Overall, 61% of all participants with AD rated their quality of life as good. **Conclusions:** Persons with an SCI are not adequately informed about AD. In most persons with AD, their symptoms are not properly recognized by healthcare providers. Our results suggest the need for more education of healthcare professionals about AD.

## 1. Introduction

In humans, a spinal cord injury (SCI) at or above the sixth thoracic (T6) segment frequently leads to a potentially life-threatening condition known as autonomic dysreflexia (AD). This syndrome is characterized by acute hypertension caused by unregulated sympathetic reflexes below the injury site, often accompanied by baroreceptor-mediated bradycardia, which temporarily regulates blood pressure [1,2,3,4]. This dangerous condition can be triggered by stimuli from the skin or internal organs below the level of the lesion [1,2,3]. As such, removing this stimulus and rapidly managing arterial hypertension is imperative for adequate treatment [1].

AD is most commonly found in persons who have an SCI at the level of T6 or higher, but can also be found in individuals with lesions as low as T10 [5]. The traumatic SCI is the most common cause of AD and other causes included vascular pathology, neoplasms, degenerative diseases, and inflammatory diseases [1,2,3]. Any painful or irritating stimuli below the level of injury may cause AD but bladder and bowel problems are the most common [1,2,3,4,5,6].

Bradycardia and sudden increase in blood pressure is the most common manifestation of AD. Accompanying symptoms include a strong headache, profuse sweating, and redness of the skin above the level of injury, and cold and pale skin below the level of the injury. As defined above, in the acute phase, AD can lead to uncontrolled arterial hypertension and hypertensive crisis, which can become life-threatening [1,2,7]. Chronically, repeated bouts of AD can result in cardiac dysfunction. For this reason, it is essential that individuals with an SCI, their caregivers, and healthcare professionals are adequately informed about AD.

Approximately, 200,000 persons are living with a chronic SCI in the United States. Healthcare costs for these individuals represent a significant economic load for the country, not to mention the physiological, psychological, and social consequences for these people [8]. Currently, Croatia has approximately 3500 individuals with a traumatic SCI [9]. The incidence of traumatic SCI is 20/106 inhabitants, e.g., approximately 100 new individuals each year [10]. Approximately 52% of persons have tetraplegia, and most of them are 16–30 years old [9]. Given the high number of persons with tetraplegia in Croatia, the likelihood of encountering episodes of AD during their lifetime is significantly increased.

The National Spinal Cord Injury Statistical Center in the USA presented that 17% of individuals with an SCI have at least one episode of AD [11]. The prevalence rate of AD is between 48 and 90% in persons with an SCI above T6 suggesting that the higher level of the lesion is accompanied with a higher probability of AD [11,12,13]. These data suggested that AD is a common clinical syndrome affecting persons with an SCI, and it must be recognized and properly treated.

The only Croatian study known by the authors clearly indicates that the knowledge about AD is insufficient among healthcare providers [7]. Adequate educational measures at the national level must be undertaken. The influence of educational interventions on professionals’ knowledge and changes in practice behavior may be enhanced with a focus on outcomes that are perceived as serious, such as the consequences of the improper management of AD [14].

Our previous study examined the knowledge of AD among nursing and physiotherapy students in two counties in Croatia [7]. The results revealed a significant gap in awareness and understanding of the clinical implications of AD among nurses and physiotherapists in these two counties, and we believe that these results could be representative for the whole country. The aim of this study was to build on this foundation and investigate the experience and knowledge of AD among individuals with SCI in Croatia. We hypothesized that the knowledge about AD was inadequate among persons with SCI and that they are not well informed about this potentially life-threatening condition.

## 2. Materials and Methods

### 2.1. Patient Characteristics and Clinical Data

The investigation was performed using an online survey. Participants included individuals living with chronic SCI throughout Croatia. An invitation for participation in the study was electronically sent to local associations of people with SCI and was presented on their online portals. Invitations for recruitment were sent to major associations of SCI individuals in Croatia including the Association of Paraplegics and Tetraplegics of Istra County (www.upitiz.hr, accessed on 10 January 2025, 29 members at the time of the study), the Association of Spinal Cord Injured Individuals Karlovac (13 members), the Spinal Cord Injury Association of Zagreb (www.soz.hr, accessed on 10 January 2025, 198 members), the Association of Spinal Cord Injury Individuals of Splitsko-Dalmatinska County (51 members), the Association of Paraplegics and Tetraplegics of Osječko-Baranjska County (47 members), and the Association of Individuals with Spinal Cord Injury Karoca Rijeka (31 members). During recruitment, potential participants were informed about the survey and voluntarily consented to participate in the study. The survey was completed online and responses were securely stored in a protected web database. The survey was opened from 1 June 2016 to 31 December 2017. After the survey was closed, the authors checked all of the completed questionnaires and did not find duplicate data (individuals).

The questionnaire (see Supplementary Materials) was developed by individuals (authors) with professional, scientific, and/or personal experience of AD. The author’s expertise was used to create this original questionnaire. The questionnaire was written in the Croatian language and was identical for all participants. The survey consisted of three sections: (i) a general, demographic section, (ii) a second section with specific questions about AD, and (iii) a third section on self-assessment and quality of life. The AD-focused survey included 23 questions of various types including case studies, multiple choice, and matching questions aimed at assessing participants’ experience with AD. After completing the survey, the data were analyzed.

Participants were divided into two groups: (i) those with an SCI at T6 and above and (ii) those with an SCI at T7 and below. Analyzing anamnestic data of persons with an SCI lesion at T6 and above with or without self-reported symptoms of AD, investigators divided them into groups with or without AD.

Clinical and demographic data are reported as median values with interquartile range (IQR), or proportions, as appropriate. Comparisons between groups were performed using the Wilcoxon rank sum test, Pearson’s Chi-squared test, or Fisher’s exact test. The main comparisons of interest included the association between the level of SCI and (a) knowledge of AD and AD associated treatments, (b) the methods used and the extent of difficulty with urinary bladder voiding, and (c) quality of life in individuals with an SCI. The statistical analysis was performed using RStudio Integrated Development Environment for R (version 2024.4.2.764), Posit Software, PBC, Boston, MA, USA. Statistical significance was set at *p* < 0.05.

### 2.2. Ethical Approval

The investigation was approved by the Ethical Committee for Biomedical Investigations of Faculty of Medicine, University of Rijeka (003-08/16-01/13, date: 13 April 2016). All the data were collected according to ethical and bioethical principles. The survey was anonymous and the collected data were protected. All participants gave their consent prior to enrolment into the study.

## 3. Results

A total of 140 individuals participated in the survey with 70 completing the entire questionnaire. The exact reason why 70 participants do not finish the survey is not known. Among the 70 responders, 51 were male and 19 were female (Table 1). In total, 41 participants had an SCI at T6 and higher while 29 had an SCI at T7 or below. The average age was 40.6 years (range from 13 to 68 years). The causes of SCI were trauma (59 individuals), tumor (3 individuals), degenerative disease (3 individuals), hereditary disease (2 individuals), spina bifida with meningomyelocele (2 individuals), and 1 case of infection.

Of the 70 individuals with an SCI, 35 (50%) reported being familiar with AD. The most common sources of information were the Internet (10 individuals), physicians (8 individuals), printed material such as books, journals, and brochures (7 individuals), nursing staff (4 individuals), other persons with an SCI (3 individuals), and other sources (3 individuals). Among those who were unfamiliar with AD, a great majority (34/35 = 97.1%) expressed the desire to learn more about the condition. Patients with a high-level SCI reported more difficulty with bladder voiding than those with a lower SCI, and intermittent self-catheterization was the most used voiding technique in both groups. Additionally, individuals with an SCI below T6 reported a slightly better self-assessed quality of life compared to those with a high-level SCI.

Using anamnestic data, individuals with an SCI at T6 and above (*n* = 41) with or without self-reported symptoms were divided into two groups: (i) those with AD (33 individuals) and (ii) those without AD (8 individuals). The cause of SCI in AD individuals was trauma in 30 (91%) persons and other causes (tumor, hereditary disease or degenerative disease) in 3 (9%) persons. Individuals with AD reported experiencing between 1 and 13 symptoms. The most frequently reported symptom was a headache (70%), followed by high muscle tone (48%) and high blood pressure combined with bradycardia (45%) (Table 2).

Most participants experienced fewer than 10 AD episodes per month, and in 70% of individuals the first onset of AD happened during the first five years after the lesion occurred (Table 3). In 21 (64%) participants, the primary trigger of AD were issues with the urinary bladder (catheter blockage, urinary tract infection, or inability to urinate). Other primary cause of AD included problems with the large intestine (hard stool, constipation, infection, or flatulence) in 6 (18%) persons, skin problems (irritation below the level of injury such as pressure or cuts, decubitus wound, nail ingrowth, sunburn or hot water, or tight clothing) in 5 (15%) persons, and other reasons (menstrual cramps, sexual activity, labor and delivery, medical tests such as cystoscopy or gynecological examination, bone fracture, stress, or medications) in 1 (3%) person. In most individuals (55%), symptoms were not recognized by the healthcare providers. The individuals with AD were dissatisfied with the information about AD they received from doctors or nurses. Despite that, 61% of all participants with AD rated their quality of life as good.

## 4. Discussion

This study for the first time investigated the knowledge about AD among a population of patients with an SCI in Croatia. Persons enrolled in the present study are relatively young, predominantly male, and mostly had a traumatic cause of SCI (84.3%). Of the 70 individuals with an SCI included in the study, 35 (50%) reported being knowledgeable about AD. The results achieved support the importance of modern media for sharing knowledge. Among the 35 persons who were unfamiliar with AD at the time of the survey, the vast majority (34/35 = 97.1%) expressed a desire to learn more about the condition.

In our cohort, in agreement with previous studies, the primary cause of AD was related to problems with the urinary bladder (64% of patients), mainly due to inadequate bladder emptying [15,16,17]. For individuals with an SCI, the close monitoring of bladder function is of the utmost importance.

A 2004 national survey in the USA which included 681 individuals with an SCI examined the self-reported importance of various physical functions in enhancing the quality of life for individuals with an SCI [8]. Regaining arm and hand function was identified as the highest priority for individuals with tetraplegia, while regaining sexual function was the highest priority for those with paraplegia. Improving bladder and bowel function was a shared priority for both injury groups. In fact, some participants ranked the recovery of bladder/bowel function and prevention of AD over regaining walking movements, highlighting the importance of addressing AD for this patient population [8].

There are multiple factors that influence quality of life in individuals with an SCI. The majority of individuals experience bowel dysfunction, and bowel care can be disabling, with a profoundly negative impact on quality of life. Bowel dysfunction has detrimental impacts on physical health, emotional wellbeing, and social function that results in lower satisfaction in this patient population. The effect of SCI on bowel function was rated as significantly worse than its effects on sex (*p* = 0.024), the bladder (*p* < 0.0001), pain (*p* = 0.013), spasticity (*p* < 0.0001), using a wheelchair (*p* < 0.0001), or skin issues (*p* < 0.0001) [18]. Priorities for bowel care routines should be to shorten the time to complete, maximize independence, and reduce the risk of fecal incontinence, constipation, and bowel-related AD [19]. In a survey including 287 individuals with an SCI, severe AD (*p* = 0.04) and a longer duration of bowel care (*p* < 0.001) were associated with a lower quality of life [18].

Not all individuals with an SCI at or above the T6 will develop AD, with the prevalence reported between 48% and 91% [20]. In our cohort, 80.5% of individuals with an SCI at T6 and above developed symptoms suggesting AD. This discrepancy could be related to differences in the completeness of the SCI, the time elapsed since injury, and differences in the criteria used to confirm the presence of AD among different studies [21].

AD most often presents in the chronic phase of an SCI, with most cases first occurring 3–6 months after injury [20]. Similarly, in our study, AD onset occurred during the first five years after the lesion occurred. Clinical evidence showed that episodes of AD often occur up to 40 times per day [20,21,22]. Most episodes of AD are managed at home or in the community by the patient or their trained caregivers and can be resolved without acute medical support [23]. Patients and their caregivers will likely have experience resolving bouts of AD and should help to guide management in the acute and long-term setting [24]. On some occasions, urgent escalation requires urgent treatment by healthcare workers. Healthcare workers who do not work in specialist centers may rarely see this condition and may not be trained for its management. It is therefore imperative for medical professionals to have a clear understanding of acute AD management [23,24]. Also, acute care management is important for the education of the patient and carer. For some at risk for AD, part of their management is the prevention of AD onset. This includes a bladder, bowel, and skin integrity program [23].

In our cohort of SCI persons with symptoms suggesting AD, the most frequently reported symptom was a severe headache, followed by high muscle tone and high blood pressure accompanied by bradycardia. In another survey, the most commonly reported symptoms of AD during the bowel care routine were goosebumps (52%), spasms (51%), flushing (49%), sweating (49%), general unwellness (43%), and headache (38%) [18]. In the above-mentioned study, more intense symptoms of AD were noticed in younger individuals (*p* = 0.009), more difficulties with the bowel care routine (*p* < 0.001), a longer time to complete bowel care (*p* = 0.0018), an increased frequency of fecal incontinence (*p* < 0.0075), and a worse QoL (*p* < 0.001) [18].

The results of the present study could serve as a basis for better management of these specific populations. Since the number of SCI persons has increased and will increase in the future, it is very important that healthcare professionals be educated about secondary complications including AD which frequently arise in this specific group of patients. Additionally, educational institutions and professional organizations must recognize this problem and implement appropriate educational platforms in the mandatory curriculum. Patient education is also an important component of the long-term management of AD, enabling the patient to act promptly to avoid complications. Furthermore, patients with recurrent AD should be taught self-management techniques such as bladder/bowel emptying and should be provided with an alert card to present when necessary. This will enable healthcare providers unfamiliar with this condition to act rapidly and effectively. AD can be easily missed by medical staff unfamiliar with this condition. Patient and healthcare provider education and a thorough evaluation are essential for the diagnosis and management of AD [17,25].

Better education and the provision of more quality information are necessary for patients with an SCI. Informational programs for individuals with an SCI and AD can be found at different online sources, such as the Paralyzed Veterans of America, the Christopher and Dana Reeve Foundation, the National Health System in the United Kingdom, and the International Spinal Cord Society [26,27,28,29]. These programs are open and provide scientific-based information about AD. In Croatia and Southeast Europe, such programs need to be established.

## 5. Limitations and Future Directions

The present study was conducted as an online survey and while it offered advantages such as accessibility and the comfort of completing it at home, it also had limitations including challenges in accurately classifying AD based on anamnestic data and a high number of incomplete questionnaires. We must be aware that some individuals with an SCI have limited access to the internet and social media and could not participate in this study. This could lead to the potential underrepresentation of some individuals not affiliated with social groups (e.g., older individuals or those struggling to complete the questionnaire). Also, individuals were classified as those with/without AD based on their answers in the survey. This is a potential bias because without direct contact with the SCI individuals, it is sometimes difficult to determine whether the AD episodes indeed occurred. Also, the overall number of participants included in this survey was relatively small which precluded further statistical analyses from being carried out. Specific domains of QoL were not investigated in the present study. Future studies should address these important considerations.

## 6. Conclusions

Croatian persons with an SCI are not adequately informed about AD and, in most instances, AD symptoms are not properly recognized by healthcare workers. Our results suggest the need for more education of healthcare professionals about AD.

## Figures and Tables

**Table 1:** Demographic and clinical characteristics of the spinal cord injury cohort (*n* = 70).

Characteristic	*n*	Above T6 *n* = 41 ^1^	Below T6 *n* = 29 ^1^	*p*-Value ^2^
Age (years)	70	38 (33, 48)	39 (31, 53)	0.8
Gender	70			0.088
Female		8 (20%)	11 (38%)	
Male		33 (80%)	18 (62%)	
Etiology of lesion	70			0.2
Non-traumatic		4 (9.8%)	7 (24%)	
Traumatic		37 (90%)	22 (76%)	
Knowledge of AD?	70			0.2
No		18 (44%)	17 (59%)	
Yes		23 (56%)	12 (41%)	
Knowledge about AD treatments	70			0.3
No		25 (61%)	21 (72%)	
Yes		16 (39%)	8 (28%)	
Difficulty voiding	70			0.042 *
Non-significant		14 (34%)	17 (59%)	
Significant		27 (66%)	12 (41%)	
Assistance during voiding	70			0.019 *
Intermittent self-catheterization		15 (37%)	17 (59%)	
Suprapubic catheter		2 (4.9%)	0 (0%)	
Bladder tapping		13 (32%)	10 (34%)	
Urethral indwelling catheter		11 (27%)	1 (3.4%)	
Urostomy		0 (0%)	1 (3.4%)	
Quality of life	70			0.071
Bad		9 (22%)	2 (6.9%)	
Intermediate		9 (22%)	13 (45%)	
Good		23 (56%)	14 (48%)	

^1^ Median (IQR) or *n* (%); ^2^ Wilcoxon rank sum test; Pearson’s Chi-squared test; Fisher’s exact test, * *p* < 0.05.

**Table 2:** Proportion of AD suggesting symptoms in patients with a spinal cord injury at T6 or above (*n* = 33).

Symptoms	*n*	Symptoms Proportion
Severe (throbbing) headache	23	0.70
High muscle tone	16	0.48
High blood pressure with slow heartbeat	15	0.45
Convulsion (cramps)	13	0.39
Feelings of fear and restlessness	12	0.36
Piloerection	12	0.36
Bristling hair, severe itching on the scalp	10	0.30
Nasal congestion	9	0.27
Red spots on the chest	9	0.27
Nausea	8	0.24
Spotty or blurred vision	8	0.24
Shivering (without fever)	7	0.21
Redness of the face and shoulders	4	0.12

**Table 3:** Clinical characteristic of the patients with autonomic dysreflexia (AD).

Characteristic	*n* = 33 ^1^
Annual incidence of AD	
0–10	10 (30%)
11–50	11 (33%)
>50	12 (36%)
Monthly incidence of AD	
≥10	9 (27%)
<10	24 (73%)
Onset of AD symptoms after lesion	
0–5 years	23 (70%)
6–10 years	4 (12%)
>10 years	6 (18%)
Primary cause of AD	
Urinary bladder	21 (64%)
Other than urinary bladder	12 (36%)
Recognition of AD symptoms	
Not recognized	18 (55%)
Recognized by the healthcare workers	8 (24%)
They were told by the individual	7 (21.0%)
Voiding difficulty	
Mild	11 (33%)
Intermediate	9 (27%)
Severe	13 (39%)
Satisfaction with AD related information from doctors	
Not satisfied	28 (85%)
Satisfied	5 (15%)
Satisfaction with AD related information from nursing staff	
Not satisfied	28 (85%)
Satisfied	5 (15%)
Satisfaction with AD treatment	
Not satisfied	29 (88%)
Satisfied	4 (12%)
Quality of life	
Bad	7 (21%)
Intermediate	6 (18%)
Good	20 (61%)

^1^ *n* (%).

## Data Availability

The data presented in this study are available on request from the corresponding author. Due to privacy and ethical restrictions, the data contain personal patient information, including names, and therefore are not publicly available.

## Supplementary Materials

The following supporting information can be downloaded at https://www.mdpi.com/article/10.3390/jcm14030670/s1, Survey of individuals with high spinal cord damage.
