# Supplementary material for: On-Line Survey About Autonomic Dysreflexia in Individuals with Spinal Cord Injury in Croatia
Source: J Clin Med. 2025 Jan 21;14(3):670. doi: 10.3390/jcm14030670 (PMC11818679; doi:10.3390/jcm14030670)
Supplement: Supplementary file 1 [file jcm-14-00670-s001.zip › jcm-3387107-supplementary.pdf]

SURVEY OF INDIVIDUALS WITH HIGH SPINAL CORD DAMAGE

In 10–30% of people with spinal cord damage above the tenth thoracic vertebra (at the level of the lower ribs), a serious set of potentially life-threatening symptoms called autonomic dysreflexia occurs. Please complete this survey in order for us to gain insight into autonomic dysreflexia in the Republic of Croatia. The survey contains 23 questions.

1. Year of birth (choose from the drop-down list): _____

2. Gender: M F

3. When was the damage to the spinal cord sustained (year of diagnosis; choose from the drop-down list):

4. Cause of spinal cord damage (one answer):

a) Trauma.

b) Tumor.

c) Degenerative diseases.

d) Hereditary diseases.

e) Other (please specify) ____

5. What is your level of spinal cord injury (select from the drop-down list)?

6. Do you know what autonomic dysreflexia is (also called hyperreflexia, sympathetic storm...)? Yes No

7. If YES, from whom did you learn what autonomic dysreflexia is (one answer):

a) Healthcare providers—doctors.

b) Healthcare providers—nurses.

c) Printed media (books, brochures, magazines).

d) Internet.

e) Other patients.

f) Other sources (specify) ____

8. If you do NOT know what autonomic dysreflexia is, would you like to learn more about this condition that can potentially endanger your life? YES NO

9. People who are treated for autonomic dysreflexia report having some of the health problems listed below. Circle the symptoms (up to 3 symptoms) that occur to you most often.

Symptoms:

- High blood pressure with simultaneous slow heartbeat (bradycardia).
- Severe throbbing headache.
- Spotty or blurred vision.
- Highly increased muscle tone (increased muscle tension).
- Convulsions (cramps).
- Vomiting.
- Feeling of fear and restlessness.
- Goosebumps above the level of damage.
- Redness of the face and shoulders.

- Sudden and strong sweating above the level of damage.

- Congestion (stuffiness) of the nose.

- Shaking (without temperature).

10. How many times a year do you have symptoms of autonomic dysreflexia (select a number from the drop-down list)?

11. How many times a month do you have autonomic dysreflexia symptoms? (choose a number from the drop-down list)?

12. How long after the spinal cord damage did you start experiencing the symptoms listed under Question 9 (previous page)? Number of years (choose from the drop-down list)_______________

13. If you have/had the above symptoms, what most often caused them (one answer)?

a) Problems with the urinary bladder (catheter blockage, urinary tract infection, inability to urinate).

b) Problems with the large intestine (hard stool, constipation, infection, flatulence).

c) Skin problems (irritation below the level of injury such as pressure or cuts, decubitus wound, nail ingrowth, sunburn or hot water, tight clothing).

d) Other (menstrual cramps, sexual activity, labor and delivery, medical tests such as cystoscopy or gynecological examination, bone fracture, stress, medications).

14. Do you know how autonomic dysreflexia is treated? YES NO

15. Did your healthcare workers themselves recognize that it was autonomic dysreflexia, did you tell them this beforehand, or did you complain about symptoms that, despite this, were not recognized (one answer)?

a) Healthcare workers recognized by themselves.

b) They were told.

c) I complained about the signs, but they were not recognized.

16. How do you rate your difficulty urinating (circle one answer)?

Disturbances are extremely rare and insignificant	Disturbances are not frequent and are of minor importance	Disturbances are relatively frequent and significant	Disturbances are frequent and urination is difficult	Disturbances are very frequent and urination is very difficult
1	2	3	4	5

17. When urinating, how do you help yourself (one answer)?

a) Suprapubic procedure (pressing or tapping the lower part of the abdomen).

b) Intermittent (self)catheterization.

c) You have a suprapubic catheter placed.

d) You have a urethral catheter in place.

e) You have a urostomy.

18. How often have you noticed signs of autonomic dysreflexia when urinating (or catheterizing) (one answer)?

Very rarely	Rarely	Sometimes	Often	Always
1	2	3	4	5

19. How satisfied are you with the information about your disease (autonomic dysreflexia) received from the doctors (one answer)?

Not at all	A little	Quite a lot	A lot
1	2	3	4

20. How satisfied are you with the information about your disease (autonomic dysreflexia) that you received from the nurses/technicians (one answer)?

Not at all	A little	Quite a lot	A lot
1	2	3	4

21. How satisfied are you with the treatment of autonomic dysreflexia (one answer)?

Not at all	A little	Quite a lot	A lot
1	2	3	4

22. How would you rate the overall quality of your life (one answer)?

Very bad	Bad	Mediocre	Good	Very good
1	2	3	4	5

23. How much does autonomic dysreflexia affect the quality of your life (one answer)?

Very little	Little	Moderately	Greatly	Extremely
1	2	3	4	5

We thank you for the time and effort you invested in filling out this questionnaire!
